# Spondylocostal Dysplasia in a 7-Year-Old Sri Lankan Girl Causing Restrictive Lung Disease: A Case Report and Review of the Literature

**DOI:** 10.1155/2020/9241207

**Published:** 2020-10-06

**Authors:** Phirarthana Kamalanathan, Meranthi Fernando, Rohan Jayawardena, A. Upasena, Shaman Rajindrajith, Sachith Mettananda

**Affiliations:** ^1^Colombo North Teaching Hospital, Ragama, Sri Lanka; ^2^Department of Paediatrics, Faculty of Medicine, University of Kelaniya, Kelaniya, Sri Lanka; ^3^Department of Paediatrics, Faculty of Medicine, University of Colombo, Colombo, Sri Lanka; ^4^Lady Ridgeway Hospital for Children, Colombo, Sri Lanka

## Abstract

Spondylocostal dysplasia (SCD) is a rare costovertebral malformation characterised by short-trunk short stature. It is a recessively inherited disorder, and commonly identified disease-causing mutations are in *DLL3* gene. The reported prevalence is 1 : 200,000 worldwide, and none was reported from Sri Lanka. We report a 7-year-old Sri Lankan girl with spondylocostal dysplasia presenting with short stature and scoliosis. Disproportionate short stature was noted with short upper segment and small thoracic cavity. Skeletal survey revealed fused vertebra involving T5-T6, T9-T10, and L3-L4. Butterfly vertebrae were noted in T2, T4, T6, and T9. Diagnosis of SCD was made based on classic radiological features including vertebral fusion and rib abnormalities. Spirometry was performed due to small thoracic cavity which showed results compatible with moderate to severe restrictive lung disease. The child did not report respiratory difficulties or recurrent chest infections up to the presentation. She was referred to an orthopaedic team which recommended conservative management with close follow-up. In conclusion, spondylocostal dysplasia should be considered in short-trunk short stature with rib abnormalities in the absence of limb shortening. Appropriate treatment and follow-up for restrictive lung disease would determine the long-term outcome.

## 1. Introduction

Spondylocostal dysplasia (SCD) is a rare heritable genetic disorder characterised by malformations of the axial skeleton resulting in vertebral and rib abnormalities [1, 2]. Short stature, scoliosis, and small thoracic cavity are key clinical features. The reported prevalence worldwide is 1 : 200,000, and a higher prevalence of 1 : 12,000 is noted in Puerto Rico [3]. The disease-causing mutations are mostly observed in the *DLL3* gene [2, 4]. Autosomal dominant inheritance is rarely reported due to mutations in *TBX6* gene [2, 5]. In many occasions, none of these mutations are identified and the diagnosis is made based on classic radiological features [5]. Affected individuals sustain a varying degree of morbidity due to the expressed phenotype [2, 5]. Severely affected children might die in early infancy due to thoracic insufficiency syndrome, whilst others could survive well into adulthood [1, 2]. Progressive restrictive lung disease is observed in the survivors due to the inherent features of small thoracic cavity and scoliosis. Little is reported on SCD especially in children beyond infancy and adults, as severe phenotypes die in early infancy, whereas the diagnosis is often overlooked in survivors [2, 3, 5]. Besides, there were no cases reported from Sri Lanka. Thus, there is more to be explored on the natural history and the phenotypic variants in this rare genetic disorder.

Here, we describe a 7-year-old Sri Lankan girl with spondylocostal dysplasia who presented due to short stature and scoliosis and was found to have moderate to severe restrictive lung disease on spirometry.

## 2. Case Report

**Figure 1:**
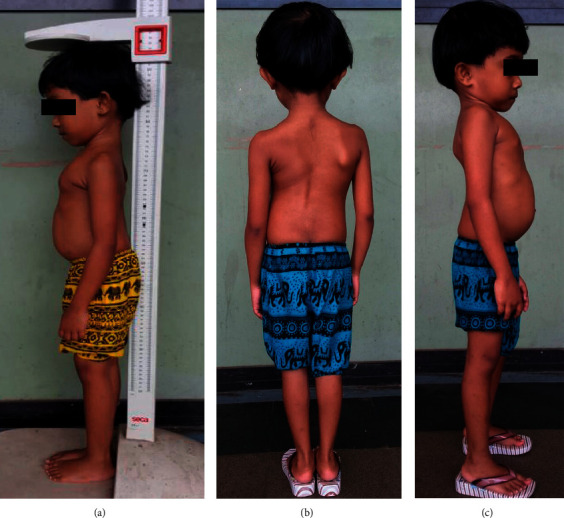
Photographs showing short stature (a), scoliosis (b), and lumbar lordosis (c).

A 7-year-old girl presented with short stature and abnormal posture. She is the only child of healthy nonconsanguineous parents from the same village. She was born at term with a birth weight of 2.5 kg, and her perinatal period was uneventful. Her early childhood development was age appropriate, and school performance was average. There were no other members of the family who had short stature or abnormal body posture. Her height was recorded as 95.5 cm which was far below the 3^rd^ percentile and the height predicted by the midparental height. The upper segment was short, and the upper-to-lower segment ratio was 0.94. Examination of the spine revealed thoracolumbar scoliosis, lumbar lordosis with a visibly small thoracic cavity, and asymmetrical rib anomalies (Figures 1(a)–1(c)). She did not have limb shortening, organomegaly, corneal clouding, or joint contractures. Rest of the physical examination was normal.

**Figure 2:**
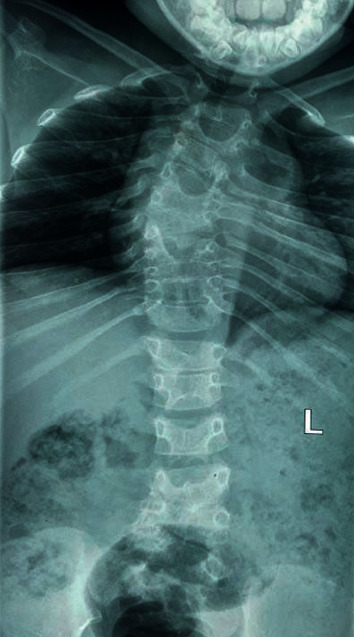
X-ray of thoracolumbar spine showing multiple fused, butterfly vertebrae and asymmetrical rib anomalies.

Basic haematological and biochemical investigations including full blood count, serum electrolytes, renal and liver function tests, serum calcium, serum inorganic phosphate, and serum alkaline phosphatase were normal. Skeletal survey revealed fused vertebra involving T5-T6, T9-T10, and L3-L4. Butterfly vertebrae were noted in T2, T4, T6, and T9. Thoracic cavity was small, and asymmetrical rib anomalies with posterior fusion of the left 6^th^ and 7^th^ ribs were noted (Figure 2).

Spirometry was performed due to small chest cavity and scoliosis. The results showed forced vital capacity (FVC) of 53%, forced expiratory volume in first second (FEV1) of 66%, and FEV1/FVC ratio of 102%, compatible with moderate to severe restrictive lung disease. However, there were no respiratory tract infections or difficulty in breathing reported in the past, and her exercise tolerance was normal.

The diagnosis of spondylocostal dysplasia was made based on short-trunk short stature, scoliosis, small thoracic cavity, and the characteristic radiological abnormalities. She was referred to a rheumatologist and to an orthopaedic surgeon for physical rehabilitation and breathing exercises and for consideration of growth plate insertion.

## 3. Discussion

We summarized a case of a 7-year-old girl from Sri Lanka who was diagnosed with spondylocostal dysplasia, a rare disease causing short trunk-short stature. Considering the age at presentation and the clinical features, it is deemed that she is having a moderately severe phenotype. However, she had scoliosis and small chest cavity causing moderate to severe restrictive lung disease. We report the key features in our case while describing the best available literature.

Short-trunk skeletal dysplasia comprises a spectrum of diseases that uses various nomenclatures in the literature [1, 2]. Spondylocostal dysplasia (SCD), spondylothoracic dysplasia (STD), and Jarcho–Levin syndrome (JLS) had been described in close association. Also, “dysplasia” and “dysostosis” are used interchangeably. JLS is a broad group that consists of short-trunk dwarfism associated with rib and vertebral anomalies. It is subdivided into STD and SCD [4]. STD is characterised by bilateral rib anomalies, whereas SCD is known to have rib anomalies in a hemithorax [4]. Solomon et al. reported that rib bifurcations, broadening, and fusion are more common in SCD than in STD in which fanlike ribs are observed [6].

The clinical phenotype of the child described in this case report demonstrates classical features of SCD that include disproportionate short stature due to a short trunk, asymmetrical rib anomalies, and scoliosis similar to previous descriptions in the literature. Her arms appeared to be relatively longer due to short chest, and the abdomen is slightly protuberant due to the lumbar lordosis. Malformations of the vertebrae in SCD could span from total fusion of the adjacent vertebrae to presence of underdeveloped, wedge shaped, or hemivertebrae [2]. This child had fused and butterfly vertebrae leading to kyphoscoliosis with hemothorax rib anomalies. Expression of the clinical phenotype is reported to be varied within the same family; nonetheless, in this case, there were no other affected family members. In severe phenotypes, thoracic insufficiency syndrome causes respiratory failure which is the commonest cause of death during infancy [1]. Recurrent respiratory tract infections have been reported in older children and adults [2]. This child did not have respiratory symptoms yet, although she was found to have moderate to severe restrictive lung disease on spirometry. Southam et al. reported a 31-year-old lady who was managed by a single physician since birth and sustained a near-normal life and employment and enjoyed gymnastics [1]. However, she experienced respiratory difficulties when she became pregnant at 18 years and required termination of pregnancy. Pochaczevsky et al. described 3 cases where generalised spina bifida vera was observed with severe shortening of the spine [7]. Diastematomyelia was reported by Kansal et al. in association with SCD referred to as JLS in an 18-month-old child who had weakness of the lower limbs [5].

Cornier et al. prospectively analysed 27 patients which was the largest cohort of patients with STD [2]. Clinical phenotype, pedigrees, and pulmonary functions were assessed [2]. Eighteen patients had succumbed to death during early infancy where the causes of death were pneumonia and respiratory insufficiency [2]. The oldest patient from that cohort was 47 years [2].

Intelligence is spared in this group of skeletal dysplasia, and average academic performance was observed in this child [1, 2, 6]. Neurological complications are rare, and we did not observe any neurological abnormalities. Pulmonary hypertension is reported due to small thoracic cavity and increased pressures; however, the child we describe did not have features to suggest increased pulmonary strain. Other reported associations of SCD comprise congenital heart defects, abdominal wall malformations, genitourinary malformations, upper limb abnormalities, neural tube defects, and congenital diaphragmatic hernia of which none was observed by us in this case [2, 8].

Turnpenny et al. reported mutations of *DLL3* as the commonest genetic defect [4, 8]. These genes are known to involve in producing proteins for NOTCH signalling pathway which is necessary for development of vertebrae and ribs [2]. Diagnosis of SCD could be based on clinical features together with classic radiological findings where presence of genetic mutations would help to subtype the disease and family screening [7]. In certain occasions, no mutations were discovered despite extensive workup [2, 8]. Availability of genetics for this rare disorder is another limitation which would preclude a genetic diagnosis in most cases. In this child, we were unable to perform genetic testing as it was not available locally and the family could not afford it from elsewhere.

Among the differential diagnoses, there are number of disorders which exhibit vertebral and rib anomalies such as camptomelic dysplasia, occulo-auriculo-vertebral spectrum, multiple pterygium syndrome, sirenomelia, Klippel-Feil syndrome, Robinow syndrome, and VACTERL syndrome [2, 7]. Casamassima-Morton-Nance syndrome is known to have spinal and rib malformations of spondylocostal dysplasia combined with urogenital abnormalities [8]. However, there were no other features or organ anomalies to suggest alternative differential diagnosis in this child.

There is no curative therapy for SCD, and the management is mainly targeted at symptomatic therapy, support, and counselling. This would best be carried out in a multidisciplinary setup in the presence of a paediatrician, orthopaedic specialists, physiotherapists, respiratory physicians, psychologists, and geneticists.

Treatment of SCD is aimed at arresting spinal curve progression and addressing the restrictive lung disease. Scoliosis is the ultimate causative factor for restrictive lung disease as it will be progressively worsened with growth. This can be achieved by addressing the thoracic insufficiency by performing multiple expansion thoracoplasties with vertical expandable prosthetic titanium rib VEPTR instrumentation (Synthes) [9, 10]. Ramirez et al. reported 20 patients with SCD who had VEPTR device with a 2-year follow-up and showed promising results [10]. In that cohort, 70% of the patients demonstrated reductions of the Cobb coronal angle and 70% had stable oxygen levels. One patient was able to bring down the overnight oxygen requirement [10]. There was no procedure that related mortality, and the authors justify the procedure comparing the higher degree of morbidity related to the disease itself. In contrast, a conservative approach against the more invasive approach of management has been discussed by Southam et al. by describing a 31-year-old who lived a near-normal life with conservative management [1]. Similarly, in our patient, the orthopaedic surgeon felt that close monitoring and conservative management should be offered at present. Chest physiotherapy had been discussed to improve long-term pulmonary function especially when there are recurrent respiratory tract infections [2]. The long-term prognosis depends on respiratory complications, while our patient had moderate to severe restrictive lung disease which will be a determining factor of her morbidity and mortality [11, 12]. Chronic back pain is another chronic medical issue encountered in this group. The 31-year-old lady who was described by Southam et al. required naproxen for exacerbations of chronic back pain [1].

Despite number of morbidities being linked to SCD, affected individuals survive into adulthood and live a near-normal life. Nevertheless, this is related to the severity of the clinical phenotype [2]. We counselled this family in detail regarding the disease, its natural history, inheritance pattern, and prognosis especially with the presence of moderate to severe restrictive lung disease.

In conclusion, this story highlights the importance of considering spondylocostal dysplasia in any child with short-trunk short stature. Restrictive lung disease is the main determinant of life span and the quality of life and thus requires timely management.
